# Supplementary material for: Cryo-electron tomography structure of Arp2/3 complex in cells reveals new insights into the branch junction
Source: Nat Commun. 2020 Dec 22;11:6437. doi: 10.1038/s41467-020-20286-x (PMC7755917; doi:10.1038/s41467-020-20286-x)
Supplement: Supplementary file 1 — Supplementary Information [file 41467_2020_20286_MOESM1_ESM.pdf]

Supplementary Information

Cryo-electron tomography structure of Arp2/3 complex in cells reveals new insights into the branch junction

Florian Fäßler[1], Georgi Dimchev[1], Victor-Valentin Hodirnau[1], William Wan[2], Florian KM Schur[1*]

Affiliations:
[1] Institute of Science and Technology (IST) Austria, Klosterneuburg, Austria
[2] Department of Biochemistry and Center for Structural Biology, Vanderbilt University, Nashville, United States of America

***Correspondence to:** florian.schur@ist.ac.at

Keywords: Arp2/3 complex, actin cytoskeleton, cryo-electron tomography, image processing, structural biology

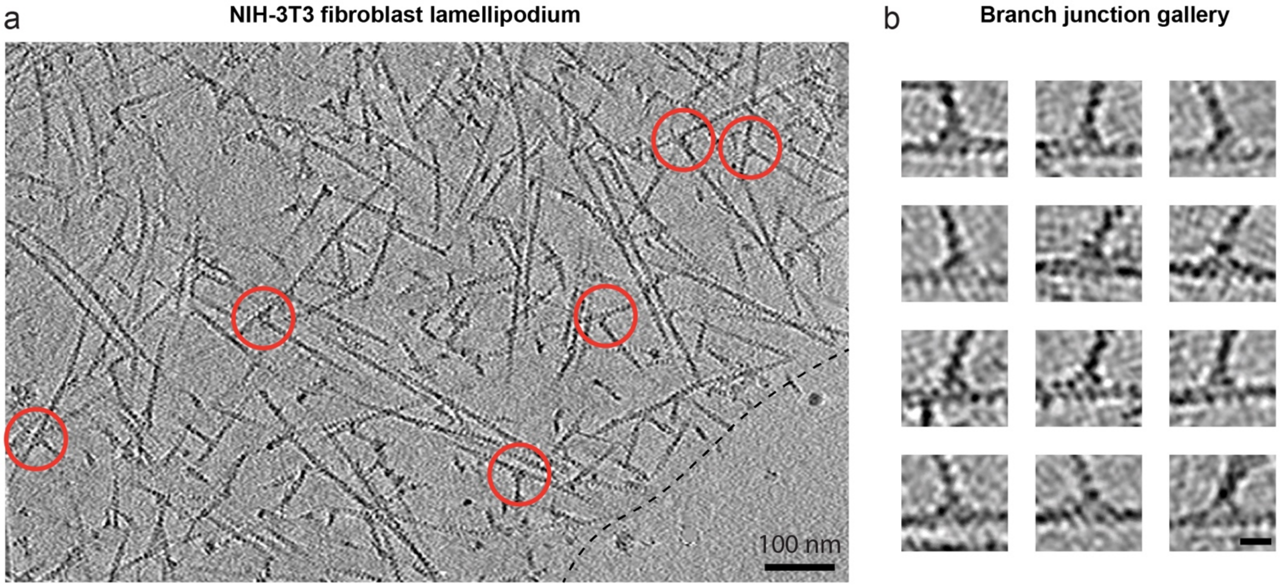

Supplementary Fig. 1.: Cryo-ET of the actin network in a NIH-3T3 fibroblast lamellipodium. (a) Computational slice through a binned, non-CTF corrected tomogram (17.096Å/px) of a NIH-3T3 fibroblast lamellipodium. Protein density is black. The dense actin network is visible and the helical appearance of individual actin filaments can be clearly appreciated. Several branch junctions are highlighted in red circles. The dashed line indicates the cell edge. Scale bar is 100nm. This slice is representative for 131 tomograms, obtained from 3 independent data acquisitions, as explained in the Methods. **(b)** Gallery of selected branch junctions. The density corresponding to the Arp2/3 complex at branch junctions is visible in the individual examples. Scale bar is 20 nm. The branch junctions in this gallery are representative for in total 14,296 branch junctions, obtained from the entire dataset of 131 tomograms.

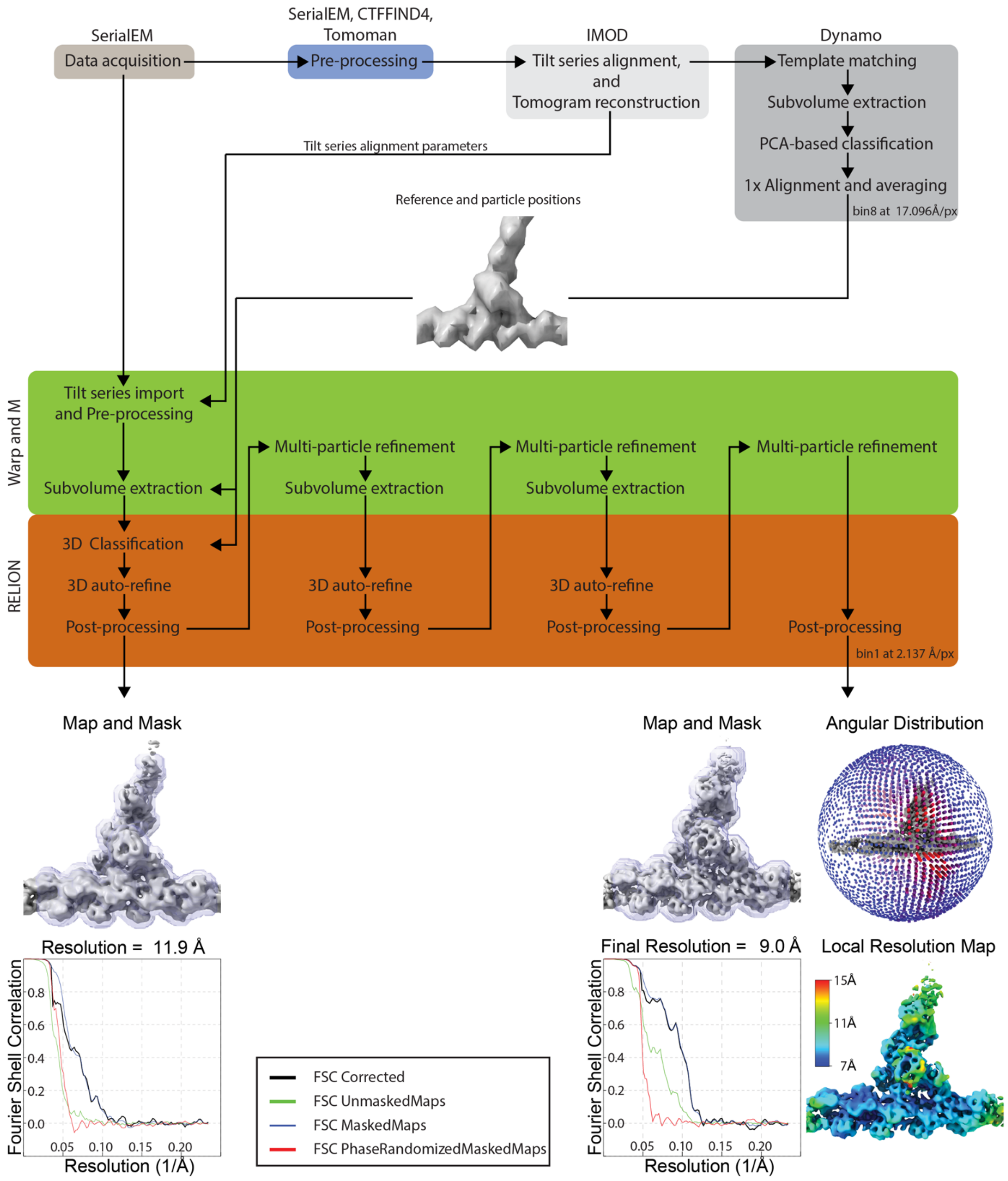

Supplementary Fig. 2.: Graphical workflow of image processing. Flow chart indicating the data processing steps involved in generating the structure of the active Arp2/3 complex within the branch junction. Colored boxes indicate usage of specific software packages. For simplicity, the procedure to produce a reference for template matching via manual picking and averaging branch junctions is not depicted here, but is described within the Methods section. In the angular distribution map less common orientations are depicted in blue while more common orientations are depicted in red. The local resolution map is colored according to the provided color key.

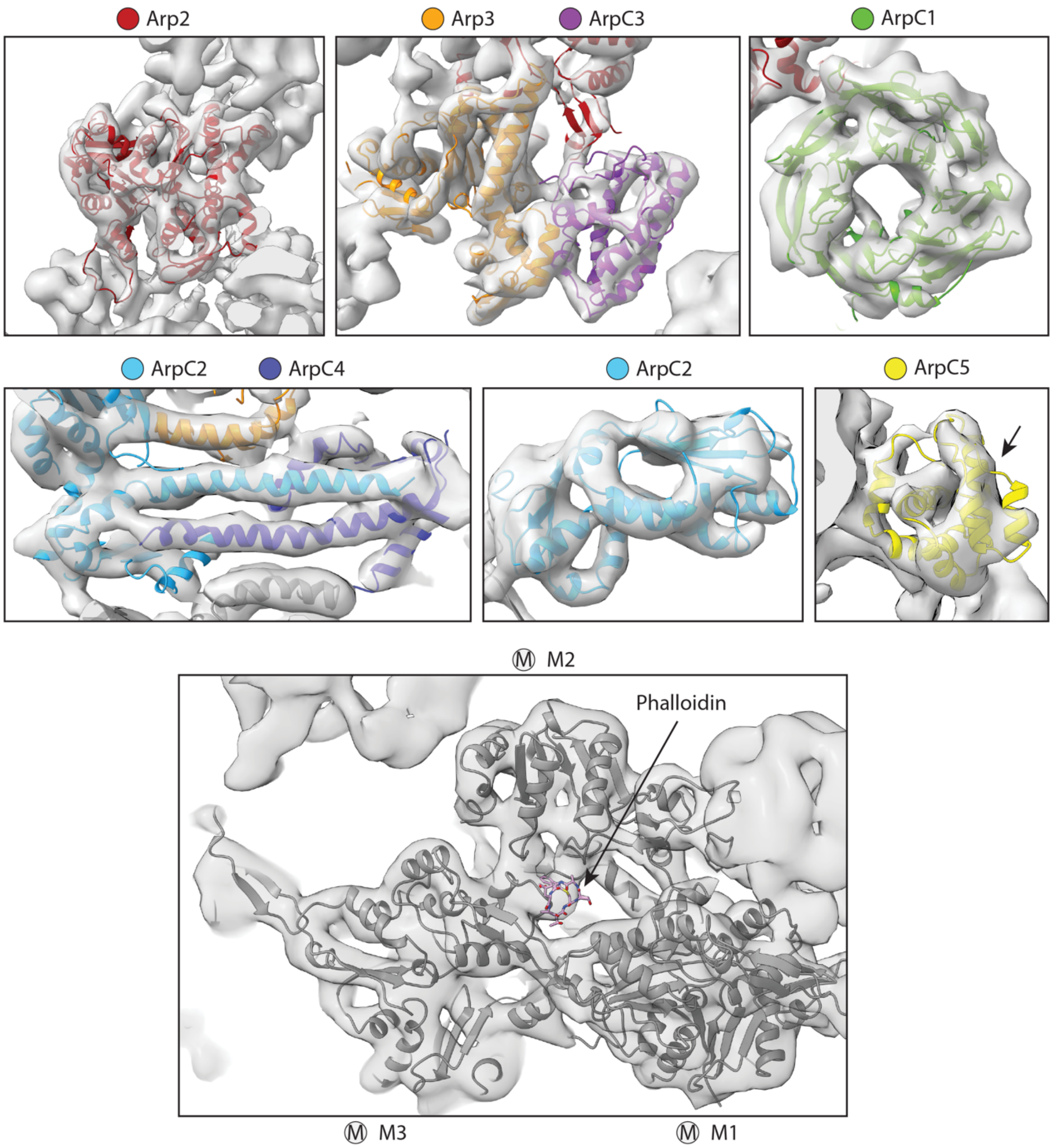

Supplementary Fig. 3.: Structural details of the actin filament Arp2/3 complex branch junction. Densities for the individual subunits of the Arp2/3 complex and the mother filament (M#) plus their fitted models are shown. Secondary structure detail (i.e. alpha helices) are clearly visualized, for example for Arp3, ArpC2 and ArpC4. The beta-propellers of ArpC1 allow unambiguous fitting of the subunit into the density of the branch junction. The increased apparent flexibility at the N-terminus of the ArpC5 helical core (annotated by an arrow) is visible, resulting in reduced density for the N-terminal helices of this subunit. The subnanometer resolution of our structure also allows to clearly visualize secondary structure details in the actin filament, further highlighted by the visibility of an additional density accommodating phalloidin (annotated by an arrow). Subunit colors are annotated and identical to the schematic guide given in Figure 1 with Arp2 being red, Arp3 orange, ArpC1 green, ArpC2 light blue, ArpC3 violet, ArpC4 dark blue and ArpC5 yellow. Actin is shown in grey.

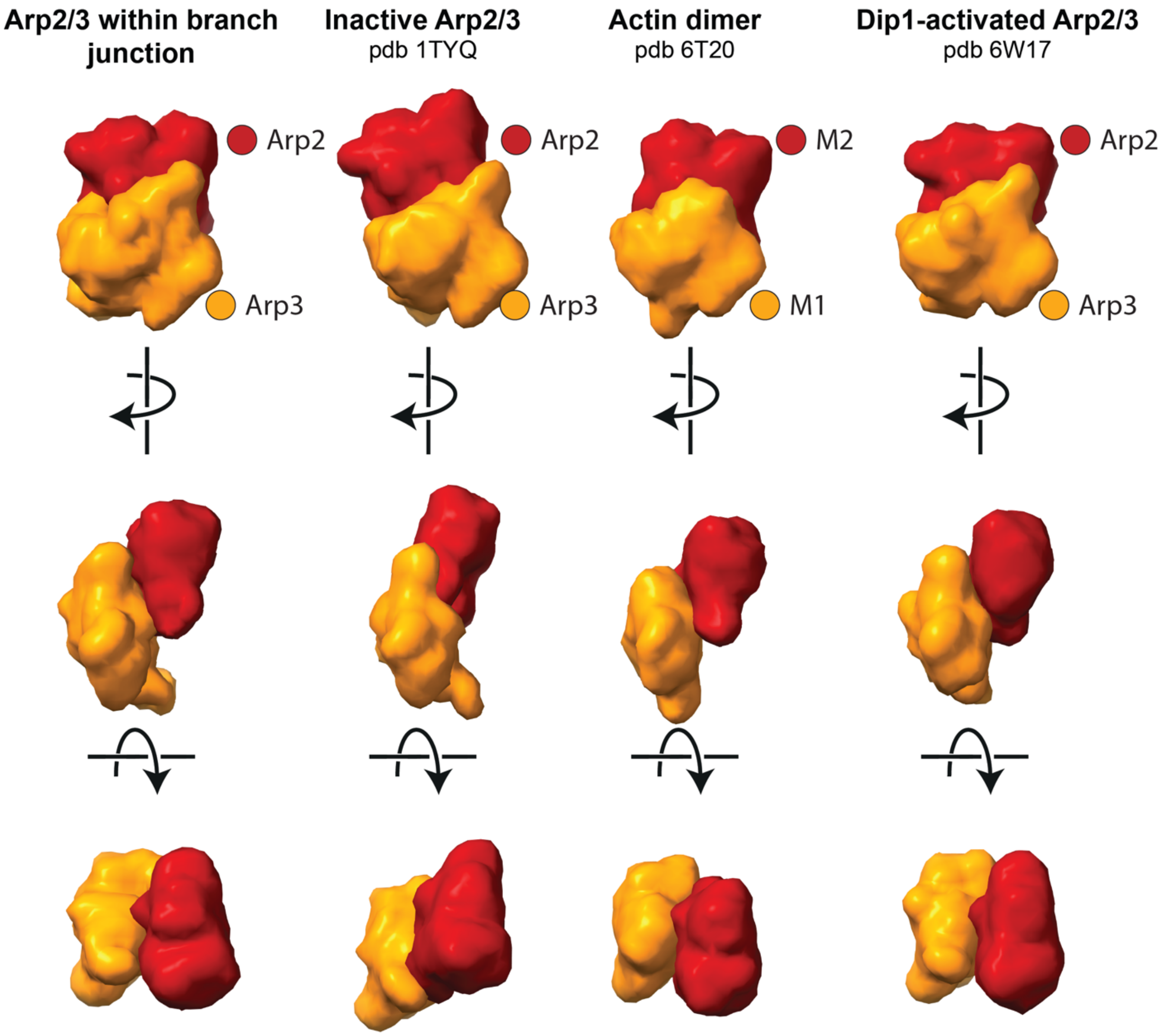

Supplementary Fig. 4.: Comparison of the short pitch actin dimer conformation to Arp2 and Arp3 in their inactive and active conformation. Density maps of molecular models of an actin dimer, or of Arp2 and Arp3 in their active and inactive conformation, respectively, filtered to a resolution of 15Å. The model of the active Arp2/3 conformation was derived from our model of the actin filament Arp2/3 complex branch junction structure in cells described in this manuscript and the *in vitro* Dip1-activated *S. pombe* Arp2/3 complex (pdb 6W17)[1]. The shown models of filamentous actin were derived from pdb 6T20[2], and for the inactive ATP-bound Arp2/3 complex from pdb 1TYQ[3]. Maps were oriented by fitting Arp3 subunits and the actin subunit M1 to each other. While Arp2 and Arp3 of the active complex adopt a similar short pitch conformation as the actin dimer, this is not the case for the Arp2 and Arp3 subunits of the inactive complex. Subunit identity is indicated by the color scheme. Arp2 and the corresponding actin subunit are shown in red and Arp3 and the corresponding subunit are shown in orange.

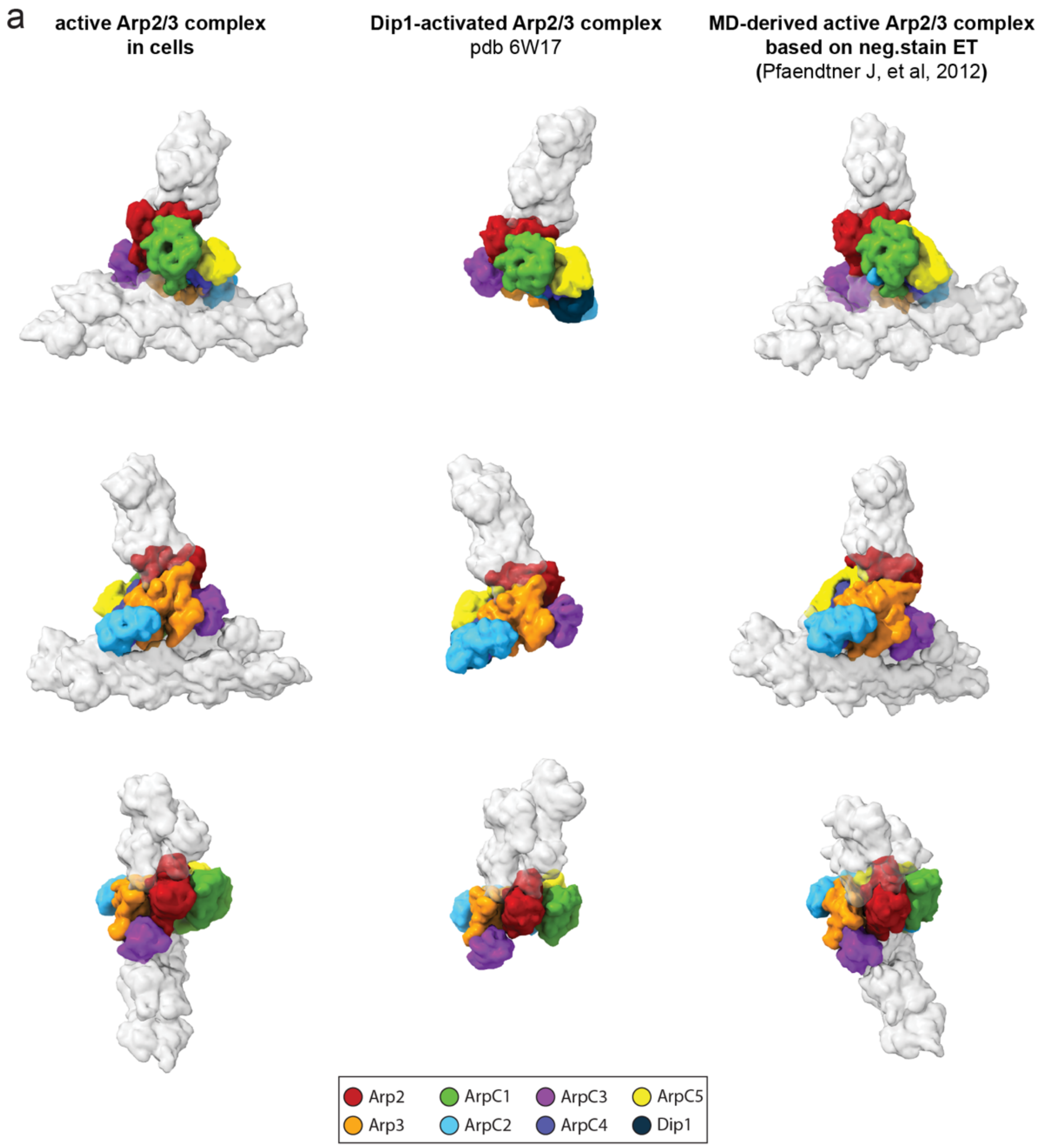

b C-alpha RMSD (Å) between subunits in active Arp2/3 complex in cells (this study) and Dip1-activated Arp2/3 complex (pdb 6W17)

subunit used for alignment	Arp2	ArpC1	ArpC4	ArpC5	Arp3	ArpC2	ArpC3
Arp2	2.4	5.7	4.6	6.8	4.7	5.6	6.8
ArpC1	5.8	4.1	5.1	4.0	9.8	10.2	15.6
ArpC4	6.4	5.3	2.3	3.9	8.1	4.9	15.9
ArpC5	8.1	6.5	4.3	2.1	10.5	10.2	11.8
Arp3	5.8	7.7	5.5	4.8	2.6	3.6	7.5
ArpC2	4.9	6.7	4.3	3.7	4.0	2.1	11.7
ArpC3	8.5	18.3	16.8	22.3	7.1	17.1	4.1

c C-alpha RMSD (Å) between subunits in active Arp2/3 complex in cells (this study) and MD-derived active conformation based on neg.stain ET

subunit used for alignment	Arp2	ArpC1	ArpC4	ArpC5	Arp3	ArpC2	ArpC3
Arp2	3.9	7.9	8.4	13.4	7.3	9.1	10.1
ArpC1	13.0	5.1	3.9	4.7	11.7	6.8	26.2
ArpC4	12.8	5.4	3.3	4.5	14.5	6.8	29.2
ArpC5	9.6	8.3	4.9	1.8	10.5	5.0	22.5
Arp3	6.8	10.1	9.9	17.6	6.5	8.8	7.4
ArpC2	11.1	8.1	5.3	6.5	10.8	3.0	23.4
ArpC3	27.1	35.9	32.4	51.3	14.3	21.8	3.2
MF	18.0	22.4	14.1	28.3	12.0	15.0	12.9

Supplementary Fig. 5.: Comparison of the active Arp2/3 complex conformation in branch junctions in cells to the *in vitro* Dip1-activated Arp2/3 complex and to a previously published MD-derived *in vitro* branch junction model. (a) Molecular models of the Arp2/3 complex in the active conformation (shown as density maps filtered to 9.5Å resolution) as observed in cells (left, this study), the *in vitro* Dip1-activated Arp2/3 complex (middle)[1], and a MD-derived active Arp2/3

complex (right)[4], which is based on a low-resolution negative stain ET reconstruction[5]. Subunit colors are annotated and identical to the schematic guide given in Figure 1, with Arp2 being red, Arp3 orange, ArpC1 green, ArpC2 light blue, ArpC3 violet, ArpC4 dark blue and ArpC5 yellow. Actin is shown in grey. The models were aligned on the ArpC2 subunits to visualize the different conformations of the Arp2/3 complex and in case for the branch junction also the varying position on the mother filament between the *in situ* and *in vitro* model. **(b-c)** RMSD values (in Å) calculated between the subunits of the three models shown in **(a)**. Rows indicate which subunit was used for aligning full models to each other, prior to measurements between individual subunits (indicated in the columns) of the different models of the active Arp2/3 complex. **(b)** RMSD values of differences between the Arp2/3 complex in branch junctions in cells and the *in vitro* Dip1-activated Arp2/3 complex. In order to calculate C-alpha RMSD values between our model and pdb 6W17 only primary structure areas are considered, in which the *B. Taurus* (as used in our model) and *S. pombe* protein sequences are in the same register, hence omitting inserts present in only one species. This comparison reveals differences between the Dip1-activated Arp2/3 complex *in vitro* and the activated state of the Arp2/3 complex in cells, in particular with respect to ArpC3. **(c)** RMSD values of differences between the Arp2/3 complex in branch junctions in cells and the MD-derived *in vitro* branch junction model.

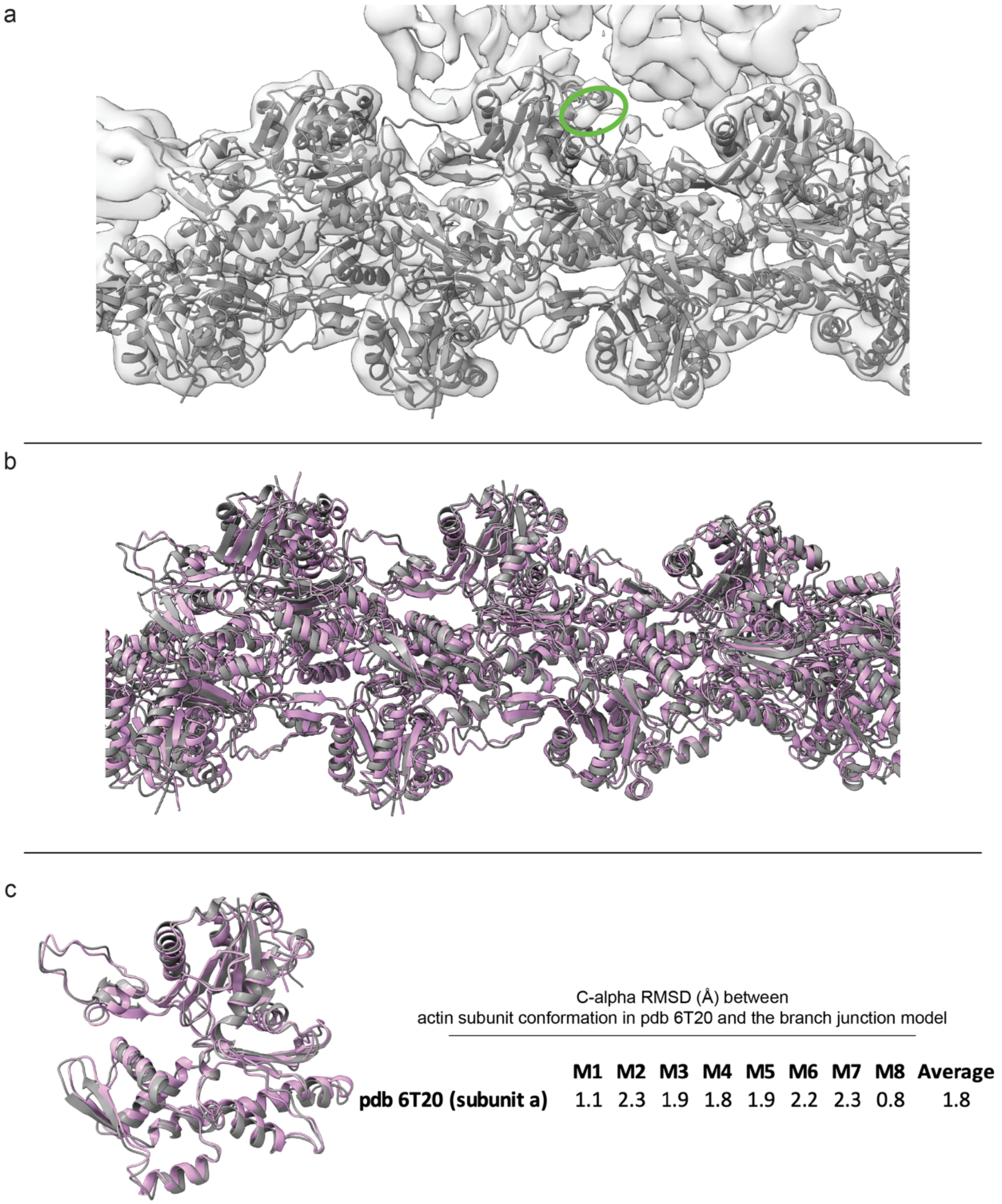

C-alpha RMSD (Å) between actin subunit conformation in pdb 6T20 and the branch junction model

	M1	M2	M3	M4	M5	M6	M7	M8	Average
**pdb 6T20 (subunit a)**	1.1	2.3	1.9	1.8	1.9	2.2	2.3	0.8	1.8

Supplementary Fig. 6.: Mother actin filament conformation in the actin-Arp2/3 complex branch junction. (a) Fit of the final model of the mother actin filament after MD-refinement into the EM density of the branch junction using ISOLDE[6]. The empty density close to M4, corresponding to the helix of ArpC1 is annotated with a green ellipsoid. **(b)** Superimposition between the starting model used for fitting (pdb 6T20, pink) and the final model after MD-refinement. Small deviations between the filament assemblies can be observed. **(c)** Superimposition between one actin subunit of pdb 6T20 (pink) and subunit M4 of the mother filament in our branch junction model. No large-scale deviations in the subunit conformation are observed. **(d)** RMSD calculations between the C-alpha atoms of one subunit of pdb 6T20 and the subunits of the mother filament. The average RMSD value is 1.8Å.

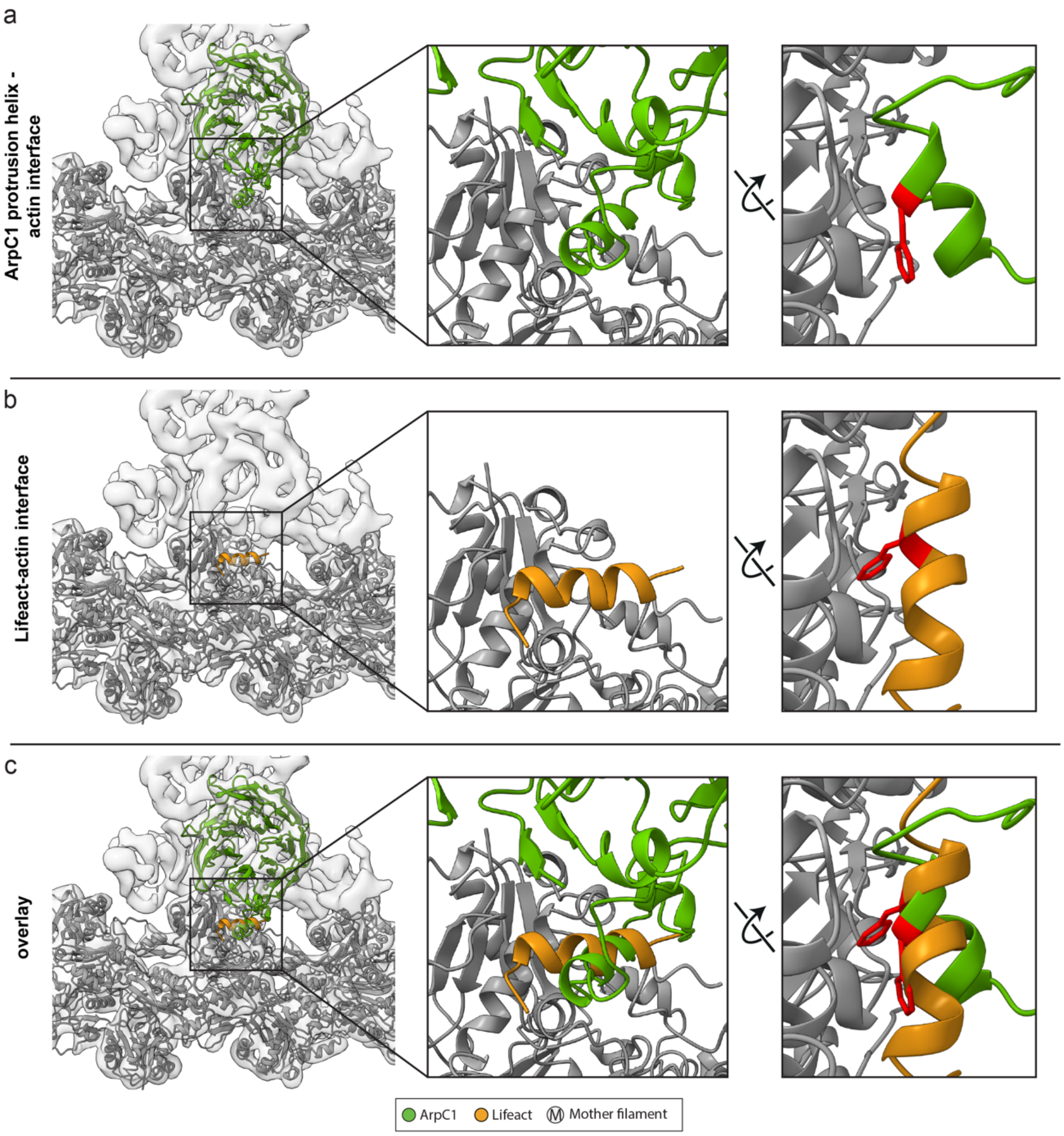

d

```
Lifeact      1    MGVADLIKKFESISKEE   17
ArpC1b     294    RGL-TARERFQNLDKKA   309
                   *:    ::*:.:.*:
```

Supplementary Fig. 7.: The ArpC1 protrusion helix and Lifeact share the same actin filament binding site

(a-c) Overview of the ArpC1 protrusion helix-actin and Lifeact-actin interface and a direct overlay of both for comparison (left). The middle panel shows magnified views of the interface. The right panel additionally shows a rotated view of the middle panel where Phe302 in ArpC1 and Phe10 in Lifeact, which can participate in the hydrophobic interface, are highlighted. ArpC1 is colored green, actin in grey and Lifeact is shown in orange. **(d)** Sequence alignment of the ArpC1 helix and Lifeact. The same residues as shown in a-c) are highlighted in red. The model of Lifeact was derived from pdb 7BTE[7]. An asterisk indicates full conservation.

Sample		NIH-3T3 fibroblast lamellipodia
Acquisition settings	Microscope	FEI Titan Krios
	Voltage (keV)	300
	Detector	Gatan BioQuantum K3
	Energy-filter	Yes
	Slit width (eV)	20
	Super-resolution mode	Yes
	Å/pixel	2.137
	Defocus range (microns)	-1.75 to -5.5
	Defocus step (microns)	0.25 to 0.5
	Acquisition scheme	-60/60°, 2°, dose-symmetric
	Total Dose (electrons/Å^2)	~170
	Dose rate (electrons/pix/sec)	19.25
	Frame number	7
	Tomogram number	131
Processing settings	Subvolumes after template matching	39,300
	Subvolumes final	14,296
	Final resolution (0.143 FSC) in Å	**9**

Table S1.
Data acquisition and image processing parameters

Subunit	pdb 1TYQ	residues added with coot	residues added from other source (source)	residues cropped compared to 1TYQ	residue stubs completed for side chains for modeling in ISOLDE
Arp2	151-345	1, 2, 3	4-150, 346-387 **(4JD2)**		27, 36, 42, 53, 106, 118, 125, 153, 173, 174, 175, 180, 181, 290, 291, 292, 294, 295, 296, 329, 330, 331, 336, 339, 340, 341, 345, 366, 380, 383, 384
Arp3	2-39, 52-353, 360-418	1, 40-51, 354-359			79, 262, 263, 264, 265
ArpC1	1-288, 319-372	289-296, 310-318	297-309 **(1k8k)**		1, 130,131
ArpC2	1-208, 217-281	209-216		282	207
ArpC3	2-150, 155-174	1, 151-154, 175-178			
ArpC4	3-168	1, 2			
ArpC5	36-151			10-27, 35	74

Table S2.
Summary of model content
The pdb files used to generate the final model of the active Arp2/3 complex are listed. Residues that had to be added or removed from the original models are indicated. Residue stubs in the original models were completed to contain their entire side chains for MD-modelling in ISOLDE.

ISOLDE Refinement	Actin filament Arp2/3 complex branch junction
Resolution of map (Å)	9
Number of Arp2/3 complex subunits/ actin monomers	7/11
All-atom clash score	3.1
Rotamer outliers	6.7%
Ramachandran outliers	2.5%
Ramachandran favoured	84.2%
Rmsd (angles, degrees)	2.209
Rmsd (bonds, Å)	0.013

Table S3.
Modelling parameters and statistics

Residues contributing to Arp2/3 - actin filament interfaces (cut-off distance: 10Å C-alpha to C-alpha atom)

Interface	Arp2/3 complex - mother filament											Arp2/3 complex - daughter filament			
Protein	Arp3	ArpC1	ArpC2	ArpC3	ArpC4	M2	M4	M5	M6	M7		Arp2	Arp3	D1	D2
UniProtKB	P61157	Q58CQ2	Q3MHR7	Q3T035	Q148J6	P68135	P68135	P68135	P68135	P68135		A7MB62	P61157	P68135	P68135
Total # of contact residues	34	11	23	7	13	4	35	8	42	10		41	36	45	17
Residue ID	Lys 38	Thr 297	Val 176	Lys 93	Met 1	Ser 350	Lys 50	Met 227	Gly 36	Tyr 198		Glu 75	Val 146	Pro 38	His 40
	Glu 39	Ala 298	Lys 179	Tyr 96	Thr 2	Thr 351	Lys 84	Ala 228	Arg 37	Ala 231		Asn 76	Leu 149	Arg 39	Gln 41
	Ser 40	Arg 299	Val 180	Thr 97	Ala 3	Gln 353	His 87	Ala 231	Pro 38	Ser 232		Met 114	Trp 153	His 40	Gly 42
	Ala 41	Glu 300	Gln 183	Ile 100	Ile 82	Gln 354	His 88	Ser 232	Arg 39	Ser 233		Asn 115	Glu 160	Gln 41	Val 43
	Lys 42	Arg 301	Glu 184	Pro 176	Glu 83		Tyr 91	Ser 233	His 40	Ser 234		Pro 116	Arg 161	Gly 42	Met 44
	Val 43	Phe 302	Glu 187	Gly 177	Lys 144		Gln 121	Ser 234	Gln 41	Ser 239		Thr 117	Thr 162	Val 43	Val 45
	Gly 44	Gln 303	Gly 188	Gln 178	Ser 147		Glu 125	Gly 268	Gly 42	Gly 245		Lys 118	Leu 163	Met 44	Met 47
	Asp 45	Asn 304	Arg 190		Glu 148		Leu 142	Pro 322	Val 43	Gln 246		Val 143	Thr 164	Val 45	Lys 61
	Gln 46	Leu 305	His 193		Lys 150		Tyr 143		Val 45	Val 247		Leu 146	Val 180	Gly 46	Gly 63
	Ala47	Asp 306	Thr 194		Leu 151		Ala 144		Gly 46	Ile 248		Gly 150	Ala 181	Met 47	Ile 64
	Gln 48	Lys 307	Gln 197		Asn 154		Ser 145		Met 47			Leu 151	Glu 182	Gly 48	Glu 205
	Arg 49		Leu 199		Ala 155		Gly 146		Gly 48			Leu 152	Gly 183	Gln 49	Leu 242
	Arg 50		Glu 204		Arg 158		Arg 147		Lys 50			Thr 153	Tyr 184	Lys 50	Pro 243
	Val 51		Pro 205				Gly 342		Asp 51			Val 169	Val 185	Lys 61	Asp 244
	Glu 68		Pro 206				Ile 345		Ser 52			Tyr 170	Pro 308	Gly 63	Gly 245
	Lys 69		Leu 207				Leu 346		Ile 64			Glu 171	Ile 309	Ile 64	Gln 246
	Pro 70		Glu 208				Ser 348		Leu 65			Gly 172	Asp 310	Leu 65	Val 247
	Thr 71		Asp 211				Leu 346		Thr 66			Phe 173	Val 311	Thr 66	
	Tyr 72		Thr 212				Ser 350		Leu 67			Ser 174	Arg 312	Ile 75	
	Ala 73		Asp 213				Thr 351		Asp 80			Leu 175	Arg 313	Asp 187	
	Thr 74		Gly 217				Phe 352		Glu 83			Pro 176	Leu 347	Tyr 188	
	Tyr 202		Asp 218				Tyr 362		Lys 84			His 177	Lys 348	Lys 191	
	Gln 205		Phe 228				Asp 363		Trp 86			Leu 178	Leu 349	Ile 192	
	Gln 206						Glu 364		His 87			Thr 179	Ser 350	Leu 193	
	Pro 216						Ala 365		Phe 90			His 269	Glu 351	Thr 194	
	Pro 217						Gly 366		Tyr 91			Asn 272	Glu 352	Glu 195	
	Glu 218						Pro 367		Asn 92			Val 273	Leu 353	Arg 196	
	Gln 219						Ser 368		Glu 93			Glu 274	Ser 354	Gly 197	
	Ser 220						Ile 369		Leu 94			Gly 275	Gly 355	Ser 199	
	Leu 221						Val 370		Arg 95			Gln 287	Arg 357	Phe 200	
	Ala 261						His 371		Val 96			Asp 290	Leu 358	Ser 239	
	Ile 262						Arg 372		Ala 97			Ile 291	Lys 359	Glu 241	
	Ser 263						Lys 373		Lys 113			Asp 292	Pro 360	Leu 242	
	Lys 264						Cys 374		Ala 114			Thr 293	Lys 361	Pro 243	
							Phe 375		Glu 117			Arg 294	Pro 362	Asp 244	
									Lys 118			Ser 295	Ile 363	Gly 245	
									Gln 121			Tyr 325		Gln 246	
									Glu 125			Arg 328		Val 247	
									Thr 126			Val 329		Ile 248	
									Glu 364			Leu 330		Thr 249	
									Ala 365			Lys 331		Ile 250	
									Gly 366					Phe 266	
														Ile 267	
														Gly 268	
														Met 269	

Table S4. Arp2/3 complex residues contacting the actin mother and daughter filaments

Summary of residues forming interactions between the Arp2/3 complex and the actin filaments, defined by a 10Å C-alpha to C-alpha distance cutoff. The UniProt identifiers for the individual proteins are given.

References:

1. Shaaban, M., Chowdhury, S. & Nolen, B. J. Cryo-EM reveals the transition of Arp2/3 complex from inactive to nucleation-competent state. *Nat. Struct. Mol. Biol.* (2020).
2. Pospich, S., Merino, F. & Raunser, S. Structural Effects and Functional Implications of Phalloidin and Jasplakinolide Binding to Actin Filaments. *Structure* **28**, 437-449.e5 (2020).
3. Nolen, B. J., Littlefield, R. S. & Pollard, T. D. Crystal structures of actin-related protein 2/3 complex with bound ATP or ADP. *Proc. Natl. Acad. Sci. U. S. A.* **101**, 15627–15632 (2004).
4. Pfaendtner, J. *et al.* Key Structural Features of the Actin Filament Arp2/3 Complex Branch Junction Revealed by Molecular Simulation. *J. Mol. Biol.* **416**, 148–161 (2012).
5. Rouiller, I. *et al.* The structural basis of actin filament branching by the Arp2/3 complex. *J. Cell Biol.* **180**, 887–895 (2008).
6. Croll, T. I. ISOLDE: a physically realistic environment for model building into low-resolution electron-density maps. *Acta Crystallogr. Sect. D* **74**, 519–530 (2018).
7. Kumari, A., Kesarwani, S., Javoor, M. G., Vinothkumar, K. R. & Sirajuddin, M. Structural insights into actin filament recognition by commonly used cellular actin markers. *EMBO J.* **39**, e104006 (2020).
