# Supplementary material for: Cryo-electron tomography structure of Arp2/3 complex in cells reveals new insights into the branch junction
Source: Nat Commun. 2020 Dec 22;11:6437. doi: 10.1038/s41467-020-20286-x (PMC7755917; doi:10.1038/s41467-020-20286-x)
Supplement: Supplementary file 8 — Description of additional supplementary files [file 41467_2020_20286_MOESM8_ESM.pdf]

Description of Additional Supplementary Information

Title: Supplementary Movie 1.

Description: Tomogram sequence showing the lamellipodial actin network in NIH-3T3 fibroblasts The area shown in this movie corresponds to the actin network shown in Supplementary Fig. 1.

Title: Supplementary Movie 2.

Description: Rotating view of the actin filament Arp2/3 complex branch junction EM density

Title: Supplementary Movie 3.

Description: Structural tour through the actin filament Arp2/3 complex branch junction

Title: Supplementary Movie 4.

Description: Transition between inactive and active state, the structure is shown from side facing the daughter filament. The movie shows the transition of the Arp2/3 complex from the inactive state (pdb 1TYQ) to its active conformation.

Title: Supplementary Movie 5.

Description: Transition between inactive and active state, the structure is shown from the side facing the mother filament. The movie shows the transition of the Arp2/3 complex from the inactive state (pdb 1TYQ) to its active conformation.
