# Supplementary material for: Cryo-electron tomography structure of Arp2/3 complex in cells reveals new insights into the branch junction
Source: Nat Commun. 2020 Dec 22;11:6437. doi: 10.1038/s41467-020-20286-x (PMC7755917; doi:10.1038/s41467-020-20286-x)
Supplement: Supplementary file 9 — Reporting Summary [file 41467_2020_20286_MOESM9_ESM.pdf]

nature research

Corresponding author(s): Florian KM Schur

Last updated by author(s): Nov 23, 2020

# Reporting Summary

Nature Research wishes to improve the reproducibility of the work that we publish. This form provides structure for consistency and transparency in reporting. For further information on Nature Research policies, see our Editorial Policies and the Editorial Policy Checklist.

## Statistics

For all statistical analyses, confirm that the following items are present in the figure legend, table legend, main text, or Methods section.

| n/a | Confirmed | |
|---|---|---|
| ☒ | ☐ | The exact sample size (*n*) for each experimental group/condition, given as a discrete number and unit of measurement |
| ☒ | ☐ | A statement on whether measurements were taken from distinct samples or whether the same sample was measured repeatedly |
| ☒ | ☐ | The statistical test(s) used AND whether they are one- or two-sided<br>*Only common tests should be described solely by name; describe more complex techniques in the Methods section.* |
| ☒ | ☐ | A description of all covariates tested |
| ☒ | ☐ | A description of any assumptions or corrections, such as tests of normality and adjustment for multiple comparisons |
| ☒ | ☐ | A full description of the statistical parameters including central tendency (e.g. means) or other basic estimates (e.g. regression coefficient) AND variation (e.g. standard deviation) or associated estimates of uncertainty (e.g. confidence intervals) |
| ☒ | ☐ | For null hypothesis testing, the test statistic (e.g. *F*, *t*, *r*) with confidence intervals, effect sizes, degrees of freedom and *P* value noted<br>*Give P values as exact values whenever suitable.* |
| ☒ | ☐ | For Bayesian analysis, information on the choice of priors and Markov chain Monte Carlo settings |
| ☒ | ☐ | For hierarchical and complex designs, identification of the appropriate level for tests and full reporting of outcomes |
| ☒ | ☐ | Estimates of effect sizes (e.g. Cohen's *d*, Pearson's *r*), indicating how they were calculated |

*Our web collection on statistics for biologists contains articles on many of the points above.*

## Software and code

Policy information about availability of computer code

| | |
|---|---|
| Data collection | DigitalMicrograph (as included in Gatan Microscopy Suite 3.3) and SerialEM (3.7) was used for tilt-series acquisition. |
| Data analysis | Preprocessing: CTFfind4, Matlab(R2019a), SerialEM (3.7), Warp(1.0.9), TOMOMAN (08042020)<br>Tilt-series alignment and tomogram reconstruction: IMOD (4.9.12), Warp(1.0.9)<br>Particle picking and extraction: IMOD (4.9.12), Dynamo(1.1.333), Warp(1.0.9)<br>Classification and subtomogram averaging: Dynamo(1.1.333), Relion(3.0.8)<br>Multi-particle refinement: M(1.0.9)<br>Model building and analysis: UCSFChimera(1.13.1), UCSF ChimeraX(0.93), ISOLDE(1.0b5)<br>Model validation: Phenix (1.18rc3-3805) |

For manuscripts utilizing custom algorithms or software that are central to the research but not yet described in published literature, software must be made available to editors and reviewers. We strongly encourage code deposition in a community repository (e.g. GitHub). See the Nature Research guidelines for submitting code & software for further information.

## Data

Policy information about availability of data

All manuscripts must include a data availability statement. This statement should provide the following information, where applicable:

- Accession codes, unique identifiers, or web links for publicly available datasets
- A list of figures that have associated raw data
- A description of any restrictions on data availability

The EM-density map of the actin-filament Arp2/3 complex branch junction and one representative tomogram have been deposited in the EMDB (https://www.ebi.ac.uk/pdbe/emdb/) under accession numbers EMD-11869 and EMD-11870, respectively. The model of the actin filament Arp2/3 complex branch junction

was deposited in the PDB (https://www.rcsb.org/) under accession code PDB 7AQK.
The data stated above is the source for figures 1-4 and supplemental figures 1-6.
The models we used for fitting into our structure (PDB 6T20 for actin, PDB 1TYQ, PDB 1K8K and PDB 4JD2 for the Arp2/3 complex) and the model of the Dip1 activated Arp2/3 complex PDB 6W17 used for comparison are available from the PDB (https://www.rcsb.org/)

# Field-specific reporting

Please select the one below that is the best fit for your research. If you are not sure, read the appropriate sections before making your selection.

☒ Life sciences ☐ Behavioural & social sciences ☐ Ecological, evolutionary & environmental sciences

For a reference copy of the document with all sections, see nature.com/documents/nr-reporting-summary-flat.pdf

# Life sciences study design

All studies must disclose on these points even when the disclosure is negative.

| | |
|---|---|
| Sample size | Sample-size calculation was not performed prior to acquisition. Sample-size was considered large enough, since processing yielded a structure with sufficient resolution for unambiguous fitting of the protein backbones. A total of 131 tilt-series were acquired for the study and 14,296 subvolumes contributed to the final structure. |
| Data exclusions | None |
| Replication | Replication is not performed in macromolecular structural studies. Methods established in the field of structural biology were performed to ensure validity of the study. Tomograms of comparable quality as shown in Supplementary Figure 1 were reconstructed from tilt-series acquired on all three cryo-ET acquisition (samples for each acquisition were prepared independently). |
| Randomization | No allocation into experimental groups was performed as functional experiments were not part of this macromolecular structural story. |
| Blinding | Blinding was not performed as functional experiments were not part of this macromolecular structural story. |

# Reporting for specific materials, systems and methods

We require information from authors about some types of materials, experimental systems and methods used in many studies. Here, indicate whether each material, system or method listed is relevant to your study. If you are not sure if a list item applies to your research, read the appropriate section before selecting a response.

## Materials & experimental systems

| n/a | Involved in the study | |
|---|---|---|
| ☒ | ☐ | Antibodies |
| ☐ | ☒ | Eukaryotic cell lines |
| ☒ | ☐ | Palaeontology and archaeology |
| ☒ | ☐ | Animals and other organisms |
| ☒ | ☐ | Human research participants |
| ☒ | ☐ | Clinical data |
| ☒ | ☐ | Dual use research of concern |

## Methods

| n/a | Involved in the study | |
|---|---|---|
| ☒ | ☐ | ChIP-seq |
| ☒ | ☐ | Flow cytometry |
| ☒ | ☐ | MRI-based neuroimaging |

## Eukaryotic cell lines

Policy information about cell lines

| | |
|---|---|
| Cell line source(s) | The Mus musculus NIH-3T3 cell line was kindly provided by Michael Sixt. Cells are available from the European Collection of Authenticated Cell Cultures (ECACC). |
| Authentication | Not performed |
| Mycoplasma contamination | The NIH-3T3 cell line was tested negative for mycoplasma contamination. |
| Commonly misidentified lines (See ICLAC register) | No commonly misidentified cell lines were used in this study. |
